# The impact of infection-derived immunity on disease dynamics

**DOI:** 10.1007/s00285-021-01681-4

**Published:** 2021-11-12

**Authors:** Adam Le, Aaron A. King, Felicia Maria G. Magpantay, Afshin Mesbahi, Pejman Rohani

**Affiliations:** 1 Department of Mathematics and Statistics, Queen’s University, Kingston, ON Canada; 2 Department of Ecology and Evolutionary Biology, Department of Mathematics and Center for the Study of Complex Systems, University of Michigan, Ann Arbor, MI USA; 3 Odum School of Ecology and Department of Infectious Diseases, University of Georgia, Athens, GA USA

**Keywords:** Mathematical epidemiology, Mathematical modeling, Infection-derived immunity, 92D30

## Abstract

When modeling infectious diseases, it is common to assume that infection-derived immunity is either (1) non-existent or (2) perfect and lifelong. However there are many diseases in which infection-derived immunity is known to be present but imperfect. There are various ways in which infection-derived immunity can fail, which can ultimately impact the probability that an individual be reinfected by the same pathogen, as well as the long-run population-level prevalence of the pathogen. Here we discuss seven different models of imperfect infection-derived immunity, including waning, leaky and all-or-nothing immunity. For each model we derive the probability that an infected individual becomes reinfected during their lifetime, given that the system is at endemic equilibrium. This can be thought of as the impact that each of these infection-derived immunity failures have on reinfection. This measure is useful because it provides us with a way to compare different modes of failure of infection-derived immunity.

## Introduction

The *SEIS* (Susceptible-Exposed-Infected-Susceptible) and *SEIR* (Susceptible-Exposed-Infected-Recovered) models are two commonly used models in epidemiology that embody different assumptions regarding immunity after infection (Anderson and May 1991; Keeling and Rohani 2008). When we adopt the *SEIR* model, we assume that infection-derived immunity is perfect and lasts for life. Thus, in this case an individual who has recovered from an infection is assumed protected against the same pathogen again. Examples of such infectious diseases would include historical diseases of childhood including measles (Anderson and May 1991; Hethcote 2000) and rubella (Anderson and May 1983), where infection confers lifelong sterilizing immunity, with rare exceptions.

On the other hand, *SEIS* models reflect instances when there is no infection-derived immunity, so that once an individual recovers from an infection, they are immediately susceptible to the same pathogen with the same risk of infection as before. This assumption is consistent with what we know about several sexually transmitted diseases (STDs) such as herpes simplex A, gonorrhea, syphilis and chlamydia (Turner et al. 2006). It is also appropriate in infectious disease systems such as influenza (Dushoff et al. 2004; Hay et al. 2001), or *Streptococcus pneumoniae* (Cobey and Lipsitch 2012) where antigenic or serotype diversity mean that infection with one phenotype does not affort protection to subsequent exposures. Finally, there are infectious disease systems where convalescent immunity is insufficient to protect against future infection. For instance, clinical studies of respiratory syncytial virus (RSV) show that infections create incomplete immunity and that the risk of being reinfected is reduced by about 70% for the 6 months following the initial infection (Hall et al. 1991; Pangesti et al. 2018; Ohuma et al. 2012).

Infection-derived immunity can thus fail in different ways and each mode of failure has distinct implications for the reinfection probability of previously infected individuals and the overall dynamic behaviour of the disease system. There is a continuous and multidimensional space of models wherein the level of infection-derived immunity lies somewhere in between the *SEIS* and the *SEIR* models. Imperfect immunity in the form of lifelong partial immunity, as well as in the form of temporary fully protective immunity, have both been considered for their role in the transmission of a disease within a population (Gomes et al. 2004; Korobeinikov and Maini 2005; Melesse and Gumel 2010; Trawicki 2017; Yang and Silveria 1998). Nevertheless, there remain important knowledge gaps in our understanding of possible modes of immunity failure as there are many other variations of imperfect immunity that have yet to be considered. Here we consider seven models that contain different forms of imperfect infection-derived immunity, all of which contain the *SEIR* and *SEIS* models as limiting cases. The models we consider are: (i) the exponential waning model, (ii) the leaky model, (iii) the all-or-nothing model, (iv) the hyperexponential waning model, (v) the gamma-distributed waning model, (vi) the boosting model and (vii) the asymptomatic model. Some of these models have been studied previously (Bansal and Meyers 2012; Gomes et al. 2004; Rodrigues et al. 2009; Yang and Silveria 1998). In this paper we compare all seven models by deriving expressions for the probability that an individual is reinfected by the same pathogen within their lifetime, under the different model assumptions. This is called the *reinfection probability* and we will compare the expressions for reinfection probability across the seven models.

This paper is structured as follows: In Sect. 2 we describe and define the seven different models that we are comparing. In Sect. 3 we prove the existence and uniqueness of the a disease-free equilibrium and an endemic equilibrium for each model, and derive expressions for the force of infection at each endemic equilibrium. In Sect. 4 we discuss the stability of these equilibria. In Sect. 5 we present expressions for the reinfection probability of each model and compare these expressions. In Sect. 6 we summarize the results and discuss some possible future directions.

## Models

**Fig. 1:**
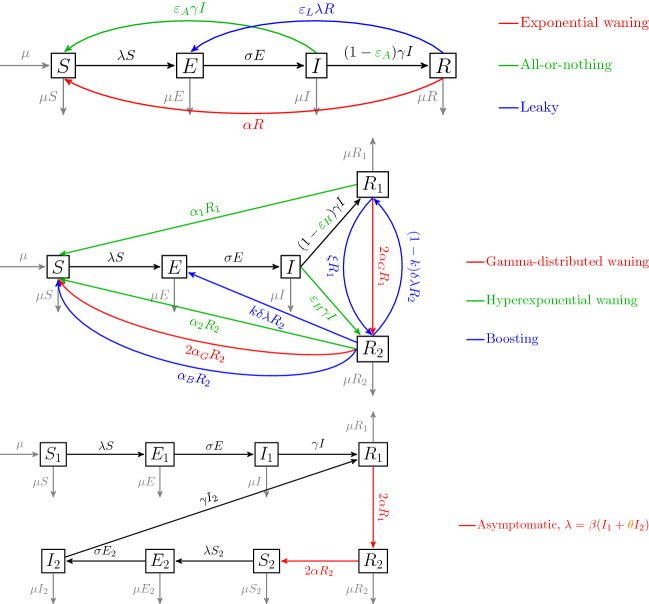
Illustration of the models

The seven different models of reinfection that we consider in this paper are illustrated in Fig. 1. The first three models (exponential waning, all-or-nothing, and leaky) require the standard four compartments: susceptible, exposed, infectious, and recovered. The next three models (gamma-distributed waning, hyperexponential waning and boosting) require a second compartment for the recovered individuals to allow for both gamma-distributed or hyperexponential waning, as well as for immune boosting. The last model requires eight different compartments to allow for a different treatment of primary infections and reinfections.

The model equations are given by (1)–(3) and descriptions of the parameters involved for the different models are summarized in Table 1. For all models, we set the compartments of the model to be given as proportions rather than numbers or densities. We also fix a birth rate of $\mu$ equal to the per capita death rate in all compartments. Thus, as long as the system is initialized properly, all compartments will remain nonnegative and the sum of all compartments will always equal 1. We assume throughout that there is a transmission rate $\beta$ between susceptible and infectious individuals, an incubation rate $\sigma$ going from an exposed to infectious compartment, and a recovery rate $\gamma$ going from an infectious compartment to another compartments.

The first three models shown in Fig. 1 are the exponential waning model, leaky and all-or-nothing immunity. The equations for these three models are given together in (1). For these three models the parameters relevant to infection-derived immunity are: the waning rate $\alpha$ for the exponential waning model, the probability of not receiving any immunity after infection (also called primary vaccine failure) $\varepsilon _A$ for the all-or-nothing model, and the “leakiness” parameter $\varepsilon _L$ for the leaky model. While it is possible to have a combination of different modes of failure in infection-derived immunity (for example, immunity that is both leaky and waning), in this paper we consider just one of the methods of failure at a time, so only one of these parameters can be nonzero at a time.

In the exponential waning model (often simply called the waning model, also called the *SEIRS* model in the literature) we assume that immunity is perfect until it disappears after an exponentially distributed amount of time. This model has been previously used to explore the resonance effects of seasonal influenza (Dushoff et al. 2004) and to quantify the evidence for loss of natural immunity to pertussis in Thailand (Blackwood et al. 2013a).

In the all-or-nothing model, we assume that while the majority of infections lead to life-long immunity, some individuals fail to develop long-term protection, as has been observed in varicella infection (Chaves et al. 2007), for example. Here, a fraction $\varepsilon _A$ of recovering individuals return immediately to the susceptible class, while the remaining $1-\varepsilon _A$ gain complete and permanent immunity.

In the leaky model, infection-derived immunity is assumed to be permanent but incompletely protective. In this model, individuals in the recovered class have a probability of infection upon exposure that is reduced by a factor $\varepsilon _L$ relative to a fully susceptible individual. This modeling assumption has been proposed in the study of influenza antigenic evolution where infection with a virus from a previous season may provide only partial immunity (Hill et al. 2019).

1 $$\begin{aligned} \frac{dS}{dt}= & {} \mu + \varepsilon _A \gamma I +\alpha R - (\lambda + \mu ) S , \nonumber \\ \frac{dE}{dt}= & {} \lambda S + \varepsilon _L \lambda R - (\sigma + \mu )E ,\nonumber \\ \frac{dI}{dt}= & {} \sigma E - (\gamma + \mu )I ,\nonumber \\ \frac{dR}{dt}= & {} (1-\varepsilon _A)\gamma I - (\varepsilon _L \lambda + \alpha + \mu ) R,\nonumber \\ \lambda= & {} \beta I. \end{aligned}$$

The equations for the first three models are identical to those of the *SEIR* model if $\varepsilon _W=\varepsilon _L=\alpha =0$. The equations become those for the *SEIS* model if we set $\varepsilon _A=1$ or $\varepsilon _L=1$ or let $\alpha \rightarrow \infty$.

The next three models presented in Fig. 1 are the hyperexponential waning model (which we often just simply call the hyperexponential model), gamma-distributed waning model (which we often just simply call the gamma-distributed model) and boosting models of infection-derived immunity. The equations for these are given in Eq. (2). The key differences between the the more commonly used basic waning model (exponential waning model, also called the *SEIRS* model) and the hyperexponential and gamma-distributed models concerns the distribution of waiting time in the immune class. The basic waning model has an exponentially distributed waning time with mean of $\frac{1}{\alpha }$ and variance $\frac{1}{\alpha ^2}$. The waning time in the gamma-distributed model is gamma-distributed (Erlang) with shape parameter of 2 has a mean of $\frac{1}{\alpha _G}$ and variance $\frac{1}{2\alpha _G^2}$. Hence, if $\alpha =\alpha _G$ this model offers a distribution with a reduced variance and as the shape parameter is increased, there is a greater central tendancy in the distribution (Keeling and Rohani 2008; Wearing et al. 2005). In contrast, the hyperexponential model offers the possibility that variance in waning times may in fact be larger than that generated by the exponential distribution. This is achieved by incorporating two compartments with associated (exponential) waning rates $\alpha _1$ and $\alpha _2$ through which fractions $\varepsilon _H$ and $1-\varepsilon _H$, respectively, of recovered individuals may regain susceptibility. Thus, a fraction $\varepsilon _H$ of recovered individuals have immunity waning at rate $\alpha _1$ and the remaining having immunity waning rate of $\alpha _2$, respectively. This results in the mean and variance of the immune period given by $\frac{\varepsilon _H}{\alpha _1}+\frac{1-\varepsilon _H}{\alpha _2}$ and $\frac{2\varepsilon _H}{\alpha _1^2}+\frac{2(1-\varepsilon _H)}{\alpha _2^2}-(\frac{\varepsilon _H}{\alpha _1}+\frac{1-\varepsilon _H}{\alpha _2})^2$, respectively. As far as we are aware, this model has not previously been explored in epidemiology.

The boosting model comprises a substantially more complicated mechanism of infection-derived immunity. In this model, it is assumed that previously infected individuals initially enter the $R_1$ compartment where they are fully protected. However, their immunity eventually wanes and they enter the $R_2$ compartment where immunity is partial with a proportion *k* of exposures in this class leading to re-infection. Partial immunity reduces the hazard of infection by the parameter $\delta \in [0,1)$. The remaining proportion $1-k$ of re-exposed individuals instead experience a boost in immunity and return to $R_1$. For individuals not re-exposed to the pathogen, immunity in $R_2$ eventually wanes and individuals become fully susceptible once again. The concept of immune boosting is further discussed in Wearing and Rohani (2009) and a models incorporating this mechanism for both infection- and vaccine-derived immunity have been fitted to pertussis time-series data in (Blackwood et al. 2013b; Lavine et al. 2013, 2011; Wearing and Rohani 2009).

2 $$\begin{aligned} \begin{aligned} \frac{dS}{dt}&= \mu +\alpha _1 R_1 +\alpha _2 R_2+ 2\alpha _G R_2 + \alpha _B R_2 - (\lambda + \mu ) S ,\\ \frac{dE}{dt}&= \lambda S + k\delta \lambda R_2 - (\sigma + \mu )E ,\\ \frac{dI}{dt}&= \sigma E - (\gamma + \mu )I ,\\ \frac{dR_1}{dt}&= (1-\varepsilon _H)\gamma I + (1-k)\delta \lambda R_2- (\alpha _1 +2\alpha _G +\xi + \mu ) R_1,\\ \frac{dR_2}{dt}&= \varepsilon _H\gamma I + 2\alpha _G R_1 +\xi R_1 -( \alpha _2+2\alpha _G+\alpha _B +\delta \lambda + \mu ) R_2,\\ \lambda&= \beta I. \end{aligned} \end{aligned}$$

The equations involved for the seventh and last model (the asymptomatic model) are given by Eq. (3). This model includes two parameters that influence immune failure and its epidemiological consequences: mean waning rate $\alpha$ and a relative infectiousness parameter $\theta$ that is equal to the reduction in infectiousness of individuals previously infected relative to naive infections. This model structure has been previously studied to examine the epidemiological consequences of repeat infections in pertussis (Blackwood et al. 2013b; Cellès et al. 2018; Wearing and Rohani 2009), rotavirus (Pitzer et al. 2009) and COVID-19 (Saad-Roy et al. 2021).

3 $$\begin{aligned} \begin{aligned} \frac{dS_1}{dt}&= \mu - (\lambda + \mu ) S_1 ,\\ \frac{dE_1}{dt}&= \lambda S_1 - (\sigma + \mu )E_1 ,\\ \frac{dI_1}{dt}&= \sigma E_1 - (\gamma + \mu )I_1 ,\\ \frac{dR_1}{dt}&= \gamma I_1 +\gamma I_2 - (2\alpha + \mu ) R_1,\\ \frac{dR_2}{dt}&= 2\alpha R_1 -( 2\alpha +\mu )R_2 \\ \frac{dS_2}{dt}&= 2\alpha R_2 - (\lambda + \mu ) S_2 ,\\ \frac{dE_2}{dt}&= \lambda S_2 - (\sigma + \mu )E_2 ,\\ \frac{dI_2}{dt}&= \sigma E_2 - (\gamma + \mu )I_2 ,\\ \lambda&= \beta (I_1+\theta I_2). \end{aligned} \end{aligned}$$

In the asymptomatic model, the $S_1$ compartment contains individuals that have never been infected before. If these individuals get exposed, they go through the first exposed class $E_1$ then first infectious class $I_1$ and we assume that these infectious individuals are *symptomatic* and infectious. Individuals that recover go to the $R_1$ then $R_2$ classes where we assume immunity is gamma-distributed. Afterwards, they become susceptible again and thus can be infected again. However, if they are re-infected we assume that they are *asymptomatic*, and possibly less infectious. For this reason, we have to have the $S_2$ class, which are susceptible individuals that have been infected before. Individuals that are reinfected then go to $E_2$ and $I_2$ classes and we assume that $I_2$ is only a fraction $\theta$, with $\theta \in [0,1]$, times as infectious as $I_1$ due to these individuals being asymptomatic. Since the waning period of immunity in (3) is gamma-distributed, the gamma-distributed model can be derived as a special case of the asymptomatic model with $\theta =1$.

**Table 1:** Description of model-specific parameters

Model	Symbol	Parameter	Allowed range	Default value in plots
All models	$\mu$	Birth and death rate	$[0,\infty )$	$\frac{1}{60}$ yr$^{-1}$
	$\sigma$	Incubation rate	$[0,\infty )$	$\frac{365}{5}$ yr$^{-1}$
	$\gamma$	Recovery rate	$[0,\infty )$	$\frac{365}{8}$ yr$^{-1}$
	$$\beta $$	Transmission rate	$$[0,\infty )$$	Varies (in yr$$^{-1})$$
Waning	$$\alpha $$	Exponential waning rate of infection-derived immunity	$$[0,\infty )$$	Varies (in yr$$^{-1})$$
All-or-nothing	$\varepsilon _A$	Probability of not receiving any immunity after infection	[0, 1)	Varies
Leaky	$\varepsilon _L$	“Leakiness,” the factor by which the probability an individual gets infected after exposure is reduced after the first infection	[0, 1)	Varies
Gamma-distributed	$$\alpha _{G}$$	Mean waning rate of immunity. Waning period has Erlang distribution with shape parameter 2 and rate $$2\alpha _{G}$$.	$$[0,\infty )$$	Varies (in yr$$^{-1})$$
Hyper-exponential	$\varepsilon _{H}$	Fraction of recovered individuals going to the $R_1$ class	[0, 1)	Varies
	$$\alpha _1$$	Exponential waning rate of infection-derived immunity of the $$R_1$$ individuals	$$[0,\infty )$$	Varies (in yr$$^{-1})$$
	$$\alpha _2$$	Exponential waning rate of infection-derived immunity of the $$R_2$$ individuals	$$[0,\alpha _1)$$	Varies (in yr$$^{-1})$$
Boosting	$\xi$	Exponential waning rate from $R_1$ to $R_2$	$[0,\infty )$	$\frac{365}{600}$ yr$^{-1}$
	$\alpha _B$	Exponential waning rate from $R_2$ to *S*	$[0,\infty )$	$\frac{365}{450}$ yr$^{-1}$
	$\delta$	Multiplier to the force of infection to obtain the “force of infection/boosting” on $R_2$	[0, 1)	0.7
	*k*	Fraction of $R_2$ experiencing “force of infection/boosting” that gets infected	[0, 1)	0.5
Asymptomatic	$\alpha$	Mean waning rate of immunity. Waning period has Erlang distribution with shape parameter 2 and rate $2\alpha$.	$[0,\infty )$	0.15 yr$^{-1}$
	$\theta$	Relative infectiousness of $I_2$ individuals relative to $I_1$	[0, 1)	0.9

The default values of the parameters used in plots (unless otherwise specified) are also given. Note that the default values were chosen so that the population is assumed to have an average lifetime of 60 years and the disease has an incubation period of 5 days and infectious period of 8 days

All model parameters are defined in Table 1. In all of the models, secondary infections do not affect the basic reproduction number (Diekmann et al. 1990; van den Driessche and Watmough 2008) of the systems. Thus for all of these models we still have

$$\begin{aligned}R_0=\frac{\beta }{\gamma +\mu }\frac{\sigma }{\sigma +\mu },\end{aligned}$$

as in the *SEIR* and *SEIS* models. This is clear because the basic reproduction number for systems like this is defined to be the spectral radius of the next-generation matrix linearized at the disease-free equilibrium (Diekmann et al. 1990; van den Driessche and Watmough 2008) and thus does not involve reinfections.

## Existence and uniqueness of equilibria

### Disease-free equilibrium

Disease-free equilibria are equilibria of each system of differential equations with the infectious compartments (*I* in the first six models, $I_1$ and $I_2$ in the last model) set to zero. Setting $I=0$ in (1) easily yields that $(S,E,I,R)=(1,0,0,0)$ is the unique disease-free equilibrium of the waning, all-or-nothing and leaky models. Similarly, setting $I=0$ in (2) yields that $(S,E,I,R_1,R_2)=(1,0,0,0,0)$ is the unique disease-free equilibrium of the gamma-distributed, hyperexponential and boosting models. Finally, setting $I_1=I_2=0$ in (3) yields that $(S_1,E_1,I_1,R_1,R_2,S_2,E_2,I_2)=(1,0,0,0,0,0,0,0)$ is the unique disease-free equilibrium of the asymptomatic model. The stability of each disease-free equilibrium depending on the values of the parameters of the models is discussed in Sect. 4.

### Endemic equilibrium

Here we prove the existence and uniqueness of the endemic equilibrium for each of the different models by proving the existence and uniqueness of a positive “force of infection” at equilibrium. The force of infection is the value denoted by $\lambda$ for each model. This is the rate at which susceptible individuals are infected and it depends on the values of the infectious compartments. We denote its fixed value at an endemic equilibrium by $\lambda _*$, solve the equilibrium equations of each model for $\lambda _*$ and show that each system yields a unique positive solution.

For some of the models it is easy to derive exact expressions for $\lambda _*$. To simplify some of our calculations, we define the following quantity,

4 $$\begin{aligned} q= \frac{\sigma }{\sigma +\mu }\frac{\gamma }{\gamma +\mu } =\text {probability of going from exposed to recovered} \end{aligned}$$

#### Exact expressions for $\lambda _*$

We first look at the expression for $\lambda _*$ for the *SEIR* model, which is a special case of (1) with $\varepsilon _L=\varepsilon _A=\alpha =0$. The force of infection at the endemic equilibrium is,

5 $$\begin{aligned} \lambda _*=\beta \Big [ \frac{\mu }{\beta }(R_{0}-1) \Big ]= \mu (R_0-1). \end{aligned}$$

The *SEIS* model is a special case of (1) with $\varepsilon _A=1$ (or $\varepsilon _L=1$ or $\alpha \rightarrow \infty$). The force of infection at this equilibrium is given by,

6 $$\begin{aligned} \lambda _*&= \beta \Big [\frac{(R_{0}-1)(\sigma +\mu )(\gamma +\mu )}{\beta (\sigma +\gamma +\mu )}\Big ]\nonumber \\&= \mu (R_{0}-1) \Big / \Big [\frac{\mu (\sigma +\gamma +\mu )}{(\sigma +\mu )(\gamma +\mu )}\Big ]\nonumber \\&= \frac{\mu (R_0-1)}{1-q}. \end{aligned}$$

For four of the seven models of imperfect immunity that we have considered, it is also possible to derive a simple form for the force of infection using an “effective” failure parameter $\varepsilon _\text {eff}$. These four models are the waning, all-or-nothing, gamma-distributed, hyperexponential models and each $\varepsilon _\text {eff}$ is defined in Table 2. Given this, the force of infection at endemic equilibrium is given by,

7 $$\begin{aligned} \lambda _*=\frac{\mu (R_0-1)}{1-q\varepsilon _\text {eff}} \end{aligned}$$

The definition of $\varepsilon _\text {eff}=\frac{\alpha }{\alpha +\mu }$ for the exponential waning model has been used before in Magpantay et al. (2014) for a model of vaccination, with a similar interpretation (the probability of immunity waning within a lifetime). There do not appear to be a simple $\varepsilon _\text {eff}$ expressions for the leaky, boosting and asymptomatic models such that we can write (7) for these models.

**Table 2:** Table of values for $\varepsilon _\text {eff}$ for the *SEIR*, *SEIS* and four others models so that the force of infection can be calculated using (7)

Model	Expression for $\varepsilon _\text {eff}$
*SEIR*	0
*SEIS*	1
Waning	$\displaystyle \frac{\alpha }{\alpha +\mu }$
All-or-nothing	$\displaystyle \varepsilon _A$
Hyperexponential	$\displaystyle (1-\varepsilon _H)\frac{\alpha _1}{\alpha _1+\mu }+ \varepsilon _H\frac{\alpha _2}{\alpha _2+\mu }$
Gamma-distributed	$\displaystyle \Big (\frac{2\alpha }{2\alpha +\mu }\Big )^2$

The endemic equilibrium force of infection for the leaky model is the positive solution to the following quadratic equation in $\lambda _*$,

8 $$\begin{aligned} \varepsilon _L(1-q)\lambda _*^2 +\mu \big [\varepsilon _L(1-R_0-q)+1]\lambda _*-\mu ^2 (R_0-1)=0. \end{aligned}$$

For this model we know that there is only one positive equilibrium and it is given by,

9 $$\begin{aligned} \lambda _* = \mu \frac{-(\varepsilon _L(1-R_0-q)+1) +\sqrt{(\varepsilon _L(1-R_0-q)-1)^2-4\varepsilon _Lq R_0}}{2\varepsilon _L(1-q)}. \end{aligned}$$

Similarly, the endemic equilibrium value of the force of infection for the boosting model can be found by finding the positive root $\lambda _*$ to the following quadratic equation:

10 $$\begin{aligned}&\big [(1-q)k\xi +\mu \big ]\delta \lambda _*^2 \nonumber \\&\quad +\big [(\xi +\mu )(\alpha +\mu )-\mu (R_0-1)(k\xi +\mu )\delta -(\alpha +\delta k\mu )q\xi \big ]\lambda _* \nonumber \\&\qquad -\mu (R_0-1)(\xi +\mu )(\alpha +\mu ) = 0. \end{aligned}$$

The exact equilibrium value for the asymptomatic model can be found by finding the positive root $\lambda _*$ to the following quadratic equation:

11 $$\begin{aligned} (1-q\varepsilon )\lambda _*^2 +\mu \big [1-(R_0-1)(1-q\varepsilon )-\theta q \varepsilon R_0\big ]\lambda _* -\mu ^2(R_0-1) = 0. \end{aligned}$$

We omit finding the explicit expressions for $\lambda _*$ in (10) and (11).

#### Properties of $\lambda _*$ using auxiliary function *f*

The explicit expressions for the force of infection at endemic equilibrium of the boosting and asymptomatic models (found by solving (10)–(11)) are long and it is not easy to show that they are unique. However we can actually prove uniqueness and derive properties of $\lambda _*$ for all the models indirectly. This is done by writing an expression involving $\lambda _*$ with the following form:

12 $$\begin{aligned} \lambda = \mu (R_0-1) + f(\lambda ). \end{aligned}$$

In Table 3 we present the forms that *f* takes for each of the different models. We omit the derivation of these forms.

**Table 3:** Expressions for the auxiliary function $f(\lambda )$ in (12)

Model	Auxiliary function $\displaystyle f(\lambda )$
*SEIR*	$\displaystyle 0$
*SEIS*	$\displaystyle q \lambda$
Waning	$\displaystyle \varepsilon _\text {eff}q \lambda$
All-or-nothing	$\displaystyle \varepsilon _\text {eff}q \lambda$
Leaky	$\displaystyle \frac{\varepsilon _L q \lambda (\lambda +\mu )}{\varepsilon _L\lambda +\mu }$
Hyperexponential	$\displaystyle \varepsilon _\text {eff}q \lambda$
Gamma-distributed	$\displaystyle \varepsilon _\text {eff}q \lambda$
Boosting	$\displaystyle \frac{(\alpha +\delta k(\lambda +\mu )) q\lambda \xi }{(\xi +\mu )(\alpha +\mu )+(k\xi +\mu )\delta \lambda }$
Asymptomatic	$\displaystyle \frac{\theta q \varepsilon \lambda R_0\mu }{(1-q\varepsilon )\lambda +\mu }$, where $\varepsilon =\Big (\frac{2\alpha }{2\alpha +\mu } \Big )^2$

Let $g(\lambda )=\lambda - \mu (R_0-1)$. From (12), the endemic equilibrium force of infection $\lambda _*$ is a solution to $g(\lambda )=f(\lambda )$. We next show that this equation always has one unique solution on $(0,\infty )$ for all the models. Clearly the solution of the *SEIR* model is $\lambda =\mu (R_0-1)$. As for the other models, if the model parameters are all nonzero, we can show that in all these cases, $f(0)=0$ and $f'(\lambda )> 0$ for all $\lambda \ge 0$. It follows from this that there is at most one positive solution to (12).

Since *g* is a simple straight line with slope equal to one, we can show that if $f''(\lambda ) \ge 0$ for all $\lambda >0$ and $f'(\infty )=\lim _{\lambda \rightarrow \infty }f'(\lambda )<1$, then there is a unique positive solution $\lambda _{*}$ to $g(\lambda _{*})=f(\lambda _{*})$ and this solution satisfies

13 $$\begin{aligned} \lambda _{*} \in \Big [\frac{\mu (R_0-1)}{1-f'(0)} ,\frac{\mu (R_0-1)}{1-f'(\infty )} \Big ] \end{aligned}$$

For instance, the auxiliary functions for the waning, all-or-nothing, hyperexponential and gamma-distributed models all have $f'(\lambda )= q \varepsilon _\text {eff}< 1$ and $f''(\lambda )=0$. Thus, the solution to $g(\lambda _{*})=f(\lambda _{*})$ satisfies $\lambda _{*} \in \Big [\frac{\mu (R_0-1)}{1-q\varepsilon _\text {eff}} ,\frac{\mu (R_0-1)}{1- q \varepsilon _\text {eff}} \Big ]$. Hence, $\lambda ^*=\frac{\mu (R_0-1)}{1-q\varepsilon _\text {eff}}$ in these cases, which is what we already found before in (7).

Expression (12) is useful to derive the range of values of the leaky model whose auxiliary function is non-linear unlike in the previous models. The first and second derivatives of the leaky model’s auxiliary function are given by

$$\begin{aligned}f'(\lambda )=\frac{\varepsilon _{L} q (\varepsilon _{L} \lambda ^{2}+ 2\mu \lambda + \mu ^{2})}{(\varepsilon _{L}\lambda +\mu )^{2}}, \qquad f''(\lambda )=\frac{2\varepsilon _{L}q\mu ^{2}(1-\varepsilon _{L})}{(\varepsilon _{L}\lambda + \mu )^{3}} \end{aligned}$$

It follows that $f'(0)=q\varepsilon _{L}$ and $f'(\infty )=q$. Both the first and second derivatives are strictly positive for all $\lambda > 0$. Therefore, the solution to $g(\lambda _{*})=f(\lambda _{*})$ must be unique and,

14 $$\begin{aligned} \lambda _{*} \in \Big (\frac{\mu (R_0-1)}{1-q\varepsilon _{L}} ,\frac{\mu (R_0-1)}{1-q} \Big ) \end{aligned}$$

This shows that the force of infection of the leaky model is greater than $\frac{\mu (R_0-1)}{1-q\varepsilon _L}$ which is the force of infection expression for waning/all-or-nothing/hyp- erexponential/gamma-distributed models with $\varepsilon _\text {eff}=\varepsilon _L$, and less than $\frac{\mu (R_0-1)}{1-q}$ which is the force of infection for the *SEIS* model.

**Fig. 2:**
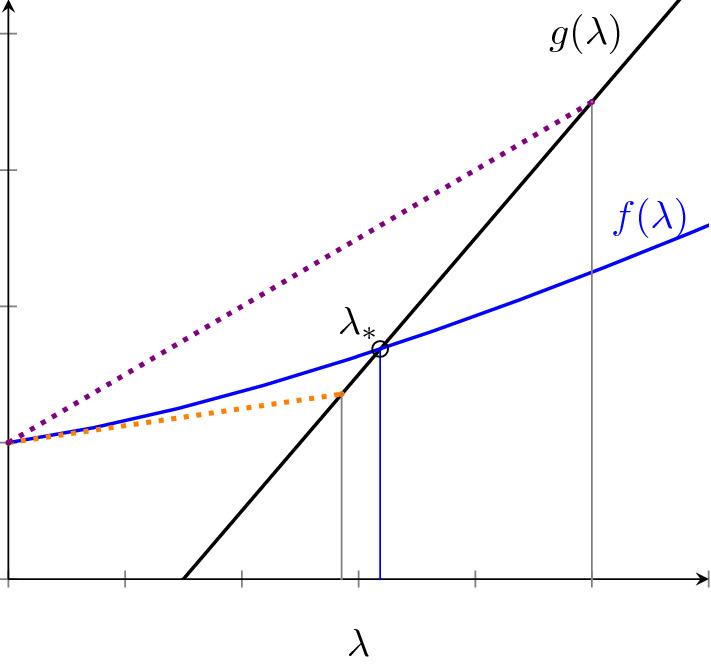
Illustration of the solution to $g(\lambda _{*})=f(\lambda _{*})$ for the leaky model. The lower bound for $\lambda _{*}$ is represented by the intersection of the dotted orange line with $g(\lambda )$. The upper bound is then represented by the intersection of the dotted purple line with $g(\lambda )$. Dotted orange and purple lines are $f'(0)\lambda$ and $f'(\infty )\lambda$ respectively (colour figure online)

Figure 2 illustrates how we found the bounds (14) to the solution of $g(\lambda _{*})=f(\lambda _{*})$ for the leaky model. The intersection between the line $g(\lambda )$ and the curve $f(\lambda )$ is the solution. The dotted orange line is $f'(0)\lambda$ and its intersection with $g(\lambda )$ establishes the lower bound in (14). Likewise, the dotted purple line is $f'(\infty )\lambda$ and its intersection with $g(\lambda )$ establishes the upper bound in (14).

For the asymptomatic model, since $f(\infty )=\lim _{\lambda \rightarrow \infty }f(\lambda )=\frac{\theta q \varepsilon R_0\mu }{1-q\varepsilon }<\infty$, there is a unique solution $\lambda _*$ that lies between the values $\mu (R_0-1)$ and $\mu (R_0-1)+f(\infty )$. The first and second derivatives of the auxiliary function are given by

$$\begin{aligned}f'(\lambda )=\frac{\theta q \varepsilon \mu ^{2} R_{0}}{((1-q\varepsilon )\lambda + \mu )^{2}}, \qquad f''(\lambda ) = \frac{-2\theta q \varepsilon \mu ^{2} R_{0}(1-q\varepsilon )}{((1-q\varepsilon )\lambda +\mu )^3}\end{aligned}$$

It follows that $f'(0)=\theta q \varepsilon R_{0}$ and $f'(\infty )=0$. Additionally, the second derivative is strictly negative for all $\lambda \ge 0$. Therefore, if $f'(0)=\theta q \varepsilon R_{0} <1$ then the solution to $g(\lambda _{*})=f(\lambda _{*})$ must satisfy

15 $$\begin{aligned} \lambda _{*} \in \Big (\frac{\mu (R_0-1)}{1-f'(\infty )},\frac{\mu (R_0-1)}{1-f'(0)} \Big ) \iff \lambda _{*} \in \Big (\mu (R_{0}-1) ,\frac{\mu (R_0-1)}{1-\theta q \varepsilon R_{0}} \Big ) \end{aligned}$$

instead of expression (13) since $f(\lambda )$ is concave down here.

**Fig. 3:**
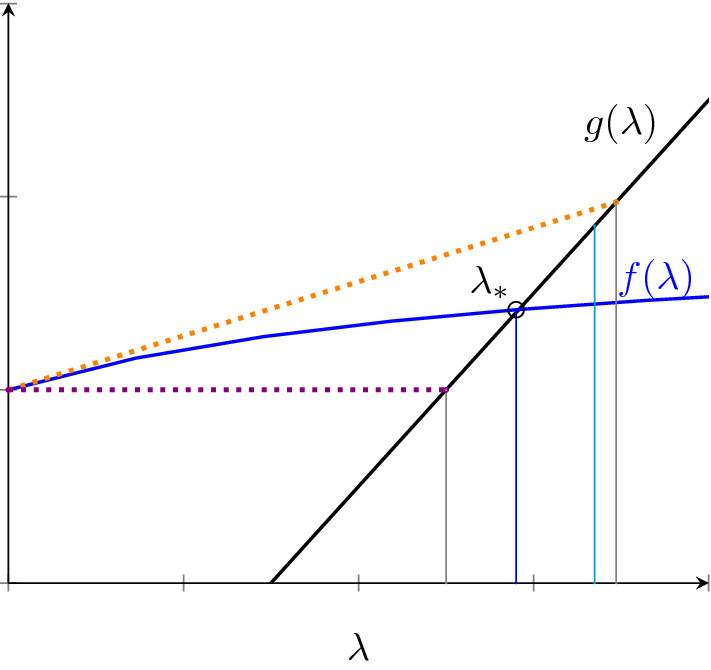
Illustration of the solution to $g(\lambda _{*})=f(\lambda _{*})$ for the asymptomatic model. The lower bound for $\lambda _{*}$ is indicated by the intersection of the purple dotted line with $g(\lambda )$. The vertical light blue line indicates $\lambda =\mu (R_{0}-1)+f(\infty )$. The upper bound of $\lambda _{*}$ is either the vertical light blue or the intersection of the dotted orange line with $g(\lambda )$ if $f'(0) < 1$. Dotted orange and purple lines are plots of the lines $f'(0)\lambda$ and $f'(\infty )\lambda$ respectively (colour figure online)

Figure 3 below illustrates how we can find bounds to $g(\lambda _{*})=f(\lambda _{*})$ for the asymptomatic model. The dotted purple line is the line $f'(\infty )\lambda$ and its intersection with $g(\lambda )$ establishes the lower bound for $\lambda _{*}$. The dotted orange line is the line $f'(0)\lambda$ for $f'(0) < 1$. Note that if $f'(0) \ge 1$ then there is no intersection between $f'(0)\lambda$ and $g(\lambda )$ at a positive $\lambda$ value. If $f'(0)\ge 1$, the upper bound of $\lambda _{*}$ is $\lambda =\mu (R_0-1)+f(\infty )$ which is represented by the vertical light blue line. If $f'(0)<1$ then the upper bound for $\lambda _{*}$ is $\min \Big \{\mu (R_{0}-1)+\frac{\theta q \varepsilon \mu R_{0}}{1-q \varepsilon },\frac{\mu (R_{0}-1)}{1-\theta q \varepsilon R_{0}}\Big \}$.

Finally, we need to consider the boosting model separately because its auxiliary function does not necessarily have a nonnegative second deriviative. The first derivative of its auxiliary function is given by

16 $$\begin{aligned} f'(\lambda )=\frac{(\xi +\mu )(\alpha +\mu )(\alpha q \xi + 2 \delta k q \lambda \xi + \delta k \mu q \xi )+(k \xi + \mu )\delta ^{2}\lambda ^{2}k q \xi }{((\xi +\mu )(\alpha +\mu )+(k\xi +\mu )\delta \lambda )^{2}} . \end{aligned}$$

From this, we get $f'(0)=\frac{q\xi (\alpha +\delta k \mu )}{(\xi +\mu )(\alpha +\mu )}<1$ and $f'(\infty )=\frac{q\xi k}{\xi k + \mu }<1$. The second derivative is then given by

17 $$\begin{aligned} f''(\lambda )=\frac{2\mu q (k\mu (1-\delta )+(1-\delta k)k\xi - \alpha (1-k))(\xi +\mu )\xi (\alpha +\mu )\delta }{((\xi +\mu )(\alpha +\mu )+(k\xi +\mu )\delta \lambda )^{3}} \end{aligned}$$

From this, we can see that $f''(\lambda )>0$ if and only if $\alpha < \frac{k\mu (1-\delta )+(1-\delta k)k\xi }{(1-k)}$. If $f''(\lambda ) > 0$ then $\lambda _* \in \Big (\frac{\mu (R_0-1)}{1-f'(0)},\frac{\mu (R_0-1)}{1-f'(\infty )}\Big )$ like in the case of the leaky model. If $f''(\lambda )<0$ then $\lambda _* \in \Big (\frac{\mu (R_0-1)}{1-f'(\infty )},\frac{\mu (R_0-1)}{1-f'(0)}\Big )$ like in the case of the asymptomatic model.

The bounds on $\lambda _*$ that we found for the leaky, boosting and asymptomatic models are summarized below in Table 4. The tightness of the bounds about the exact value of $\lambda _*$ depends on the parameters of the model. For all three models the difference between the upper and lower bounds increase with increasing value of the basic reproduction number $R_0$. If $\mu$, $\sigma$, $\gamma$ and $\beta$ are fixed (and therefore $R_0$ and *q* are also fixed), this difference still depends on the model-specific parameters. For the leaky model, the difference increase as the value of the leakiness parameter $\varepsilon _L$ increases. For the boosting model, the ratio between the two bounds depends on the value of $\frac{(\alpha +\delta k \mu )(\xi k+\mu )}{(\alpha +\mu )(\xi +\mu )k}$ (if this were equal to one then the interval for the endemic force of infection would contain just a single point). For the asymptomatic model, the bounds depend on the model-specific parameters $\theta$ and $\varepsilon$ in a more complicated manner.

**Table 4:** Interval which we can guarantee includes $\lambda _*$

Model	Interval for $\lambda _*$
Leaky	$\displaystyle \Big ( \frac{\mu (R_0-1)}{1-q\varepsilon _L} ,\frac{\mu (R_0-1)}{1-q} \Big )$
Boosting	Between $\frac{\mu (R_0-1)}{1-\frac{q\xi (\alpha +\delta k\mu )}{(\xi +\mu )(\alpha +\mu )}}$ and $\frac{\mu (R_0-1)}{1-\frac{q\xi k}{\xi k+\mu }}$
Asymptomatic	If $\theta q\varepsilon R_0>1$:
	$$\quad \displaystyle \Big (\mu (R_0-1),\mu (R_0-1) + \frac{\theta q \varepsilon R_0\mu }{1-q\varepsilon }\Big )$$
	If $\theta q\varepsilon R_0<1$:
	$$\quad \Big (\mu (R_0-1),\min \Big \{\frac{\mu (R_0-1)}{1-\theta q\varepsilon R_0 },\mu (R_0-1) + \frac{\theta q \varepsilon R_0\mu }{1-q\varepsilon }\Big \}\Big )$$
	where $\varepsilon =\Big (\frac{2\alpha }{2\alpha +\mu } \Big )^2$.

## Stability of equilibria

We use the notation ${\mathbb {R}}^n_+$ to denote the positive cone given by $\{(v_1,\dotsc ,v_n): v_1\ge 0,\dotsc v_n\ge 0\}$. Since all models were set up such that the overall birth rate equals the per capita death rate from each compartment, solutions to (1) are invariant in $\{(S,E,I,R)\in {\mathbb {R}}^4_+, S+E+I+R=1\}$, solutions to (2) are invariant in $\{(S,E,I,R_1,R_2)\in {\mathbb {R}}^5_+, S+E+I+R_1+R_2=1\}$ and solutions to (3) are invariant in $\{(S_1,E_1,I_1,R_1,R_2,S_2,E_2,I_2)\in {\mathbb {R}}^8_+, S_1+E_1+I_1+R_1+R_2+S_2+E_2+I_2=1\}$. When we discuss global asymptotic stability of the systems, we only mean this in the context of solutions initialized within each given invariant set.

### Global asymptotic stability of the disease-free equilibrium

From the general theory of compartmental epidemiological models (van den Driessche and Watmough 2002), we know that the unique endemic equilibrium of each model is at least locally asymptotically stable if the basic reproduction number $R_0<1$. To prove global asymptotic stability we use LaSalle’s Invariance Principle (LaSalle 1976; Muller and Kuttler 2015). For the waning, all-or-nothing and leaky models given by (1), we define

$$\begin{aligned}L = E +\Big (\frac{\mu +\sigma }{\sigma } \Big )I.\end{aligned}$$

The derivative of *L* along the trajectories of (1) is given by,

$$\begin{aligned} \frac{dL}{dt}&= \lambda S +\varepsilon _L \lambda R - (\sigma +\mu )E + \Big (\frac{\mu +\sigma }{\sigma } \Big )\big (\sigma E -(\gamma +\mu ) I\big ),\\&= \frac{(\mu +\gamma )(\mu +\sigma )}{\sigma }\Big [\frac{\beta }{\mu +\gamma }\frac{\sigma }{\mu +\sigma }(S+\varepsilon _L R)-1\Big ] I\\&= \frac{(\mu +\gamma )(\mu +\sigma )}{\sigma }\Big [R_0\big (S+\varepsilon _L R\big )-1\Big ] I. \end{aligned}$$

In the set $\Omega =\{(S,E,I,R)\in {\mathbb {R}}^4_+, S+E+I+R=1\}$, we know that $S+R\le 1$ which means $S+\varepsilon _L R\le 1$. If $R_0<1$ then $\frac{dL}{dt}< 0$ for as long as $I>0$. We see that $\frac{dL}{dt}=0$ only on the subset of $\Omega$ where $I=0$, and the largest invariant subset of this is $\{(S,E,I,R)\in {\mathbb {R}}^4_+, E=I=0,S+R=1\}$. Thus all trajectories starting from $\Omega$ tends towards $E=I=0$, and using this in (1) we see that, for each model, all such trajectories must approach its unique disease free equilibrium. This proves the global asymptotic stability of the unique disease-free equilibrium corresponding to each of the three models described by (1).

For the gamma-distributed, hyperexponential and boosting models given by (2), we again define $L = E +\Big (\frac{\mu +\sigma }{\sigma } \Big )I$. Taking the derivative of *L* along solutions to (2) and simplifying yields,

$$\begin{aligned}\frac{dL}{dt}= \frac{(\mu +\gamma )(\mu +\sigma )}{\sigma }\Big [R_0\big (S+k\delta R_2\big )-1\Big ] I.\end{aligned}$$

In the set $\{(S,E,I,R_1,R_2)\in {\mathbb {R}}^5_+, S+E+I+R_1+R_2=1\}$, since $k\in [0,1)$, $\delta \in [0,1)$ we must have $S+k\delta R_2\le 1$. Thus, if $R_0<1$ then $\frac{dL}{dt}<0$ for as long as $I>0$. Following the same reasoning as before, we can prove the global asymptotic stability of the disease-free equilibrium corresponding to each of the three models described by (2).

Finally, for the asymptomatic model give by (3), we define

$$\begin{aligned}L = E_1 +\Big (\frac{\mu +\sigma }{\sigma } \Big )I_1.\end{aligned}$$

Taking the derivative of *L* along solutions to (3) and simplifying yields,

$$\begin{aligned}\frac{dL}{dt}= \frac{(\mu +\gamma )(\mu +\sigma )}{\sigma }\Big [R_0 S_1-1\Big ] I.\end{aligned}$$

In the set $\Omega =\{(S_1,E_1,I_1,R_1,R_2,S_2,E_2,I_2)\in {\mathbb {R}}^8_+, S_1+E_1+I_1+R_1+R_2+S_2+E_2+I_2=1\}$ we must have $S\le 1$. Thus, if $R_0<1$ then $\frac{dL}{dt}<0$ for as long as $I_1>0$. We see that $\frac{dL}{dt}=0$ only on the subset of $\Omega$ where $I_1=0$, and the largest invariant subset of this is $\{(S_1,E_1,I_1,R_1,R_2,S_2,E_2,I_2)\in {\mathbb {R}}^8_+, E_1=I_1=E_2=I_2=0,S_1+R_1+R_2+S_2=1\}$. Thus all trajectories starting from $\Omega$ tend towards $E_1=I_1=E_2=I_2=0$, and using this in (3) we see that all such trajectories must therefore approach the unique disease free equilibrium of the asymptomatic model.

### Local asymptotic stability of the endemic equilibrium

From van den Driessche and Watmough (2002), we know that the unique endemic equilibrium of each model is at least locally asymptotically stable if $R_0>1$. Sample trajectories of these models suggest that as long as the models are initialized such that the exposed and infectious compartments are not all zero, trajectories of all the models do tend towards the endemic equilibrium if $R_0>1$. Some references confirm this “global stability” property for some special cases such as in the *SEIR*, *SEIS* and *SEIRS* (waning) models (Li and Muldowney 1995; Li and Wang 2002). There are some general methods using the LaSalle Invariance Principle and Volterra-type functions to prove global stability of the endemic equilibria of vaccination models (with imperfect vaccine-derived immunity but perfect infection-derived immunity) (Li et al. 2011), however these did not work out for our systems of equation which had no vaccination but imperfect infection-derived immunity. We do not prove global stability of the endemic equilibrium of the models in this paper, however this does not affect our results. The reinfection probabilities that we derive in the next section are computed at the endemic equilibrium which we know to at least be locally asymptotically stable.

## Reinfection probability

One way to measure the impact of imperfect infection-derived immunity is to find the probability of reinfection (the probability of returning to an exposed class within the individual’s lifetime after leaving it for the first time). This probability changes with the number of individuals infected. In this work, we evaluate this at the unique endemic equilibrium of each model.

### Definition 1

(*Reinfection probability*) Let *r* be the probability, at endemic equilibrium, that an individual goes back to being in an exposed state a second time after being in an exposed state the first time.

Since *r* is evaluated at endemic equilibrium, it depends on the value of $\lambda _*$. The value of *r* for each model is given in Table 5.

**Table 5:** Reinfection probability

Model	Reinfection probability, $\displaystyle r$
*SEIR*	0
*SEIS*	$\displaystyle \frac{q\lambda _*}{\lambda _*+\mu } = q\Big (\frac{R_0-1}{R_0-q}\Big )$
Waning	$\displaystyle \frac{q\varepsilon _\text {eff}\lambda _*}{\lambda _*+\mu } = q\varepsilon _\text {eff}\Big (\frac{R_0-1}{R_0-q\varepsilon _\text {eff}}\Big )$
All-or-nothing	$\displaystyle \frac{q\varepsilon _\text {eff}\lambda _*}{\lambda _*+\mu } = q\varepsilon _\text {eff}\Big (\frac{R_0-1}{R_0-q\varepsilon _\text {eff}}\Big )$
Leaky	$\displaystyle \frac{q\varepsilon _L\lambda _*}{\varepsilon _L\lambda _*+\mu }$
Hyperexponential	$\displaystyle \frac{q\varepsilon _\text {eff}\lambda _*}{\lambda _*+\mu } = q\varepsilon _\text {eff}\Big (\frac{R_0-1}{R_0-q\varepsilon _\text {eff}}\Big )$
Gamma-distributed	$\displaystyle \frac{q\varepsilon _\text {eff}\lambda _*}{\lambda _*+\mu } = q\varepsilon _\text {eff}\Big (\frac{R_0-1}{R_0-q\varepsilon _\text {eff}}\Big )$
Boosting	$\displaystyle \frac{q\lambda _*\xi \big (\frac{\alpha }{\lambda _*+\mu }+\delta k\big )}{(k\xi +\mu )\delta \lambda _*+(\xi +\mu )(\alpha +\mu )}$
Asymptomatic	$\displaystyle \frac{q\varepsilon \lambda _*}{\lambda _*+\mu }$ where $\varepsilon =\Big (\frac{2\alpha }{2\alpha +\mu } \Big )^2$

The expressions for *r* can be derived by following an individual in the *E* compartment and multiplying together the independent probabilities of traveling from each compartment to the next until the individual reaches the *E* compartment once again. For example, in the waning, all-or-nothing, hyperexponential and gamma-distributed models, the probability of going from *E* to *R* is given by *q*. The probability of going from *R* to *S* is given by $\varepsilon _{\text {eff}}$. Finally, the probability of going from *S* to *E* is given by $\frac{\lambda _*}{\lambda _*+\mu }$. Thus, the reinfection probability expressions for these models are given by $r=\frac{q\varepsilon _{\text {eff}}\lambda _*}{\lambda _* + \mu }$. The expressions for the *SEIS* and leaky models can be just as easily derived.

The expression for the boosting model was found by simplifying the expression:

18 $$\begin{aligned} q\frac{\xi }{\xi +\mu } \Big [\sum _{n=0}^\infty \big (\frac{\delta (1-k)\lambda }{\delta \lambda +\alpha +\mu }\frac{\xi }{\xi +\mu }\big )^n \Big ] \Big [ \frac{\alpha }{\delta \lambda +\alpha +\mu }\frac{\lambda _*}{\lambda _*+\mu } +\frac{\delta k \lambda _*}{\delta \lambda _*+\alpha +\mu } \Big ]. \end{aligned}$$

This expression accounts for all the ways that an individuals from the *E* class can go back again to the *E* class in the boosting model.

We note that the *r* values for all models except for the asymptomatic model have the form $\frac{f(\lambda _*)}{\lambda _*+\mu }$, where *f* is auxiliary function given in Table 3. The asymptomatic model does not have this form because in this case *r* is the probability of going from $E_1$ to $E_2$ instead of returning to the same *E* class. Thus in this case the reinfection probability is simply *q* times $\varepsilon =\Big (\frac{2\alpha }{2\alpha +\mu }\Big )^2$, which is the probability of going from $R_1$ to $S_2$, then times $\frac{\lambda _*}{\lambda _*+\mu }$.

**Fig. 4:**
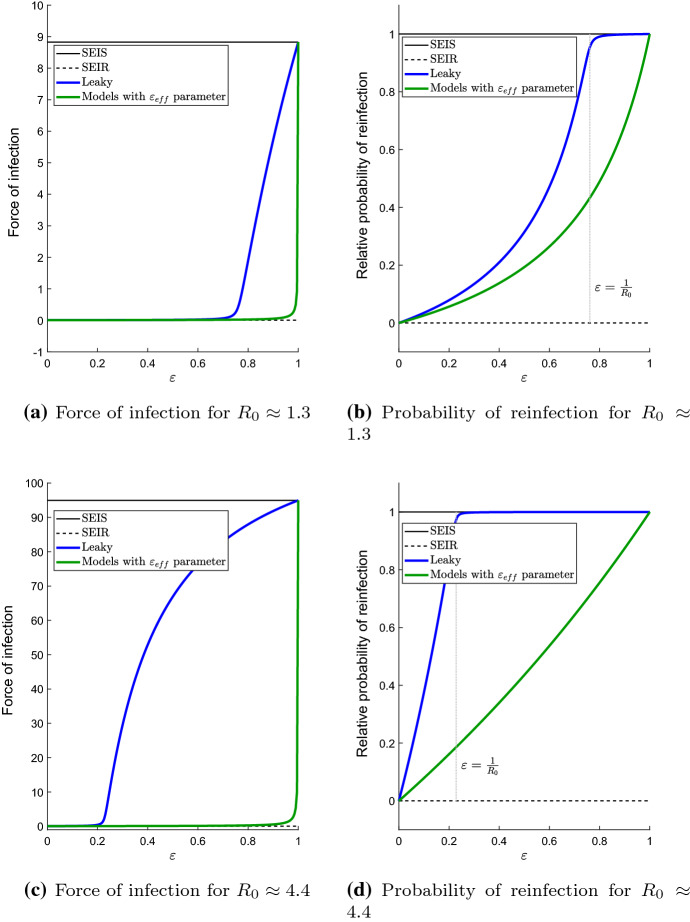
Equilibrium force of infection and relative reinfection probability of the different models at their endemic equilibria. The green curves indicate the waning, all-or-nothing, hyperexponential and gamma-distributed models. The blue curve is for the leaky model. The horizontal axis is $\varepsilon _L$ for the leaky model and $\varepsilon _\text {eff}$ for all other models. The vertical dotted grey line in (**b**) and (**d**) is where $\varepsilon = \frac{1}{R_{0}}$. Note that for the *SEIR* model, $\lambda _*>0$ (but looks close to be zero in this scale) and $r=0$. The *SEIS* model has $r\in (0,1)$. The relative reinfection probabilities are the reinfection probabilities *r* of each model divided by the *r* for the *SEIS* model. Values of $\mu$, $\sigma$ and $\gamma$ are given by the default values listed in Table 1. We used $\beta =60$ yr$^{-1}$ corresponding to $R_0\approx 1.3$ for (**a**, **b**), and $\beta =200$ yr$^{-1}$ (corresponding to $R_0\approx 4.4$) for (**c**, **d**) (colour figure online)

**Fig. 5:**
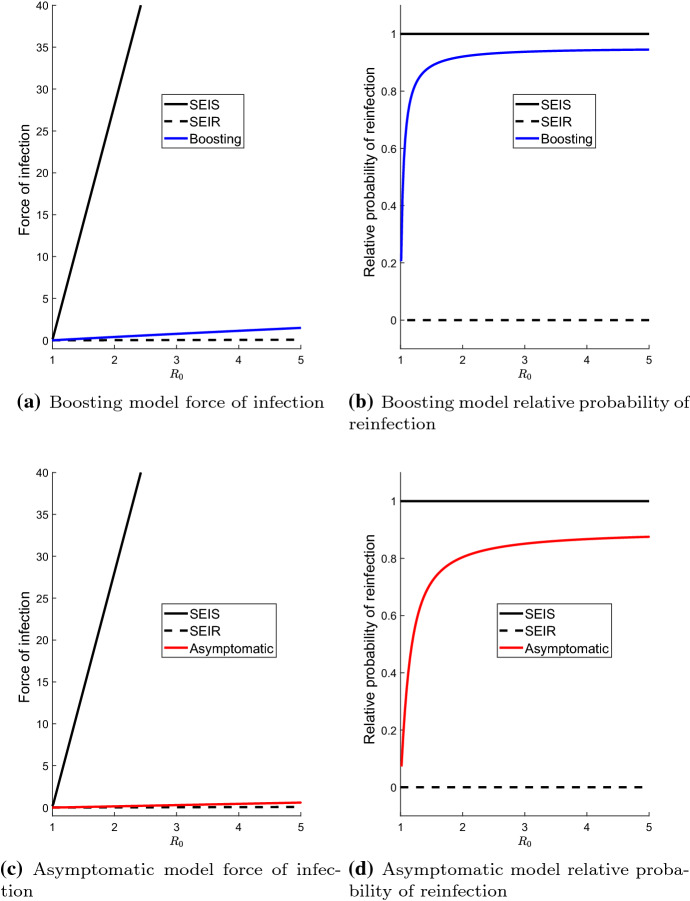
Equilibrium force of infection and relative reinfection probability of the boosting and asymptomatic models at their endemic equilibria. These are plotted as function of $R_0$. Again, note that for the *SEIR* model, $\lambda _*>0$ and $r=0$. The relative reinfection probabilities are the reinfection probabilities *r* of each model divided by the *r* for the *SEIS* model. The parameter $\beta$ is varied to change the value of $R_0$. Other parameter values are given by the default values listed in Table 1

We also computed the reduction in reinfection probability relative to the *SEIS* model. This is computed for each model by taking its *r* value and dividing it by the *r* value for the *SEIS* model with the same $R_0$. We plot the relative reduction in reinfection probabilities in Figs. 4 and 5.

## Summary and conclusions

Imperfect infection-derived immunity is a feature of many communicable diseases such as influenza, tuberculosis and potentially even COVID-19 (Roy 2020). Infection-derived immunity can wane over time, be only partially protective, completely fail in some individuals or have more complicated interactions due to immune boosting. In this study we reviewed seven compartmental disease models that feature one or more of these immunity imperfections.

To assess the impact of each models’ respective immunity failure we defined the “reinfection probability,” the probability that an individual in an exposed state goes back to the exposed state again in the future, given that the system is at endemic equilibrium. This gives a rigorous measure of how much infection-derived immunity individuals get from infection. We note that the reinfection probability for the *SEIR* model is zero and that of the *SEIS* system is between zero and one, depending on other factors such as the basic reproduction number of the system. This risk of reinfection for previously infected individuals can have implications for public health policies aimed at reaching herd immunity, such as the rollout of mass vaccination once a vaccine becomes available.

To derive the reinfection probability for each model, we first demonstrated the uniqueness of the endemic equilibrium for each model, and derived expressions and bounds for the forces of infection at the endemic equilibrium. We proved that, as expected, the force of infection for the *SEIR* and *SEIS* models are respectively the lower and upper bounds for the seven models’ force of infection. We see that the waning, all-or-nothing, hyperexponential and gamma-distributed models have a similar dynamics in which they all have an “effective failure” parameter. When these effective failure parameters are equal to 1, these models become exactly the *SEIS* model; when they are equal to 0, these models become exactly the *SEIR* model. From this, it is clear that the force of infection for these particular models lie somewhere, depending on the value of the effective failure parameter, between that of *SEIR* model’s and that of the *SEIS* model’s.

The other three models (leaky, boosting and asymptomatic models) however, have more complicated force of infection expressions that are difficult to directly compare with the *SEIS* and *SEIR* models. We therefore derived auxiliary functions to help find properties of the endemic equilibrium forces of infection for these models. These auxiliary functions reflect how much greater each models’ force of infection is to that of the *SEIR* model, where there is no possibility for reinfection. Additionally, the auxiliary functions allow us to establish the bounds for the leaky, boosting and asymptomatic models’ actual force of infection.

After deriving the endemic equilibrium forces of infection for all seven models, whether in explicit form from (7) and Table 2 or through the use of the auxiliary functions from Table 3, we derived the reinfection probability for each model. The reinfection probabilities allow us to give a measure of the impact that different types of immunity failures have on individuals. These are presented in Table 5, and we compare these to the reinfection probabilities of corresponding *SEIR* and *SEIS* models in Figs. 4 and 5. The results in Tables 2, 3, 4 and 5 summarize how the different modes by which infection-derived immunity can be lost would affect the reinfection probability and overall disease dynamics (in terms of the long-run prevalence of the disease). For example, even if the (exponential) waning and gamma-distributed waning models have the same mean waning rate ($\alpha =\alpha _G$), they would have different $\varepsilon _\text {eff}$ (see Table 2) and therefore different reinfection probabilities. Thus, even if we already know that immunity is waning (as opposed to leaky or all-or-nothing) it is not enough to know just the mean waning period of acquired immunity, we still need to know how waning occurs.

**Fig. 6:**
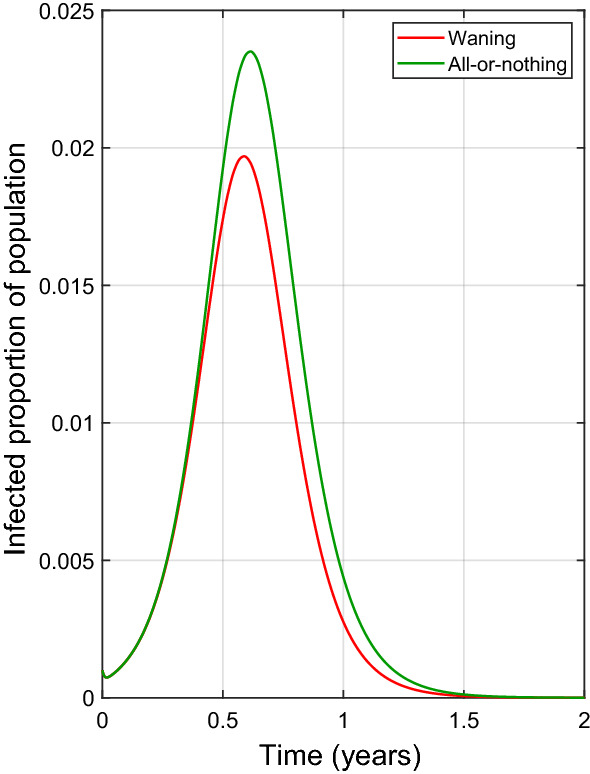
Sample trajectories of the waning and all-or-nothing models showing differences in their transient dynamics before they approach endemic equilibrium. We set $\epsilon _A=0.2$ and $\alpha =\frac{1}{240}$ yr$^{-1}$ so that $\varepsilon _\text {eff}=0.2$ for both models. The values of $\mu$, $\sigma$ and $\gamma$ are given by the default values listed in Table 1, and we used $\beta =60$ yr$^{-1}$ corresponding to $R_0\approx 1.3$. With these values, the endemic equilibrium for both models can be found using (7) and is equal to $\frac{\mu (R_0-1)}{\beta (1-q\varepsilon _\text {eff})}\approx 1.09\times 10^{-4}$. The models were initialized with $S(0)=0.999$, $I(0)=0.001$ and all other states being zero

This study opens many directions for future study. The different models with a defined $\varepsilon _\text {eff}$ (Table 2), if they are assumed to have the same basic reproduction number will have the same endemic equilibrium disease prevalence values for the same $\varepsilon _\text {eff}$ values. This suggests that it may be difficult to distinguish between these models if we are only looking at equilibria, however the models may have very different transient dynamics that show signatures of the type of infection-derived immunity. As an example, in Fig. 6 we present an illustration of the trajectories of the waning and all-or-nothing models with the same initial conditions and same values of $\mu$, $\gamma$, $\sigma$ (from Table 1), $\beta =200$ (so that $R_0\approx 1.3$) and $\varepsilon _\text {eff}=0.2$. Thus, from our results we know that these two models have the same unique endemic equilibrium which we can find from the force of infection given using (7) and Table 2. Both trajectories show that the trajectories show a peak in the infectious class before approaching the endemic equilibrium, and the peak for the all-or-nothing model is higher and later than that of the waning model.

It would also be interesting to study the leaky model further. This is the model that provides the most homogeneous sort of infection-derived protection. We note that the prevalence of infection in the leaky model is higher than the other models with a defined $\varepsilon _\text {eff}$ if $\varepsilon _L=\varepsilon _\text {eff}$ (this is evident from Table 4). We also observe that the leaky model seems to have a sudden transition from being *SEIR*-like to *SEIS*-like as $\varepsilon _L$ is increased. This transition appears to occur at the so-called “reinfection threshold” (Gomes et al. 2004, 2005).
